# Supplementary material for: Arbuscular Mycorrhizal Fungal Communities in the Soils of Desert Habitats
Source: Microorganisms. 2021 Jan 22;9(2):229. doi: 10.3390/microorganisms9020229 (PMC7912695; doi:10.3390/microorganisms9020229)
Supplement: Supplementary file 1 [file microorganisms-09-00229-s001.zip › Supplementary_rev.docx]

**Supplementary**

Table S1. Mean pairwise phylogenetic distances (mpd) between virtual taxa (VT) in different desert samples. Randomized mpd, and Z and P values were calculated using a random null model whereby mpd was calculated among randomized communities selected among VT present globally (VT in MaarjAM), VT present within continent of the sample (VT in continent) or VT present within biogeographic realm (VT in realm). Positive Z values indicate phylogenetic overdispersion; negative values indicate phylogenetic clustering. Z values < -1.96 indicated significant clustering (bold).

|  |  | Reference set: VT in Maarj*AM* | | | | |  | Reference set: VT in continent | | | | |  | Reference set: VT in realm | | | | |
| --- | --- | --- | --- | --- | --- | --- | --- | --- | --- | --- | --- | --- | --- | --- | --- | --- | --- | --- |
|  |  |  | mpd of randomized VT | |  |  |  |  | mpd of randomized VT | |  |  |  |  | mpd of randomized VT | |  |  |
|  | No of VT | mpd of observed VT | Mean | SD | Z | P value |  | mpd of observed VT | Mean | SD | Z | P value |  | mpd of observed VT | Mean | SD | Z | P value |
| AUS | 17 | 0.059 | 0.082 | 0.01 | -2.38 | **0.012** |  | 0.059 | 0.083 | 0.01 | -2.334 | **0.009** |  | 0.059 | 0.083 | 0.01 | -2.334 | **0.009** |
| ARG | 13 | 0.043 | 0.082 | 0.012 | -3.188 | **0.001** |  | 0.043 | 0.082 | 0.012 | -3.266 | **0.001** |  | 0.043 | 0.082 | 0.012 | -3.266 | **0.001** |
| ISR | 35 | 0.079 | 0.082 | 0.005 | -0.546 | 0.284 |  | 0.079 | 0.082 | 0.005 | -0.644 | 0.268 |  | 0.079 | 0.082 | 0.005 | -0.522 | 0.299 |
| KAZ | 13 | 0.049 | 0.082 | 0.012 | -2.669 | **0.001** |  | 0.049 | 0.082 | 0.012 | -2.677 | **0.003** |  | 0.049 | 0.082 | 0.013 | -2.649 | **0.002** |
| SAU | 3 | 0.057 | 0.082 | 0.031 | -0.82 | 0.239 |  | 0.057 | 0.082 | 0.03 | -0.821 | 0.237 |  | 0.057 | 0.081 | 0.03 | -0.795 | 0.231 |
| USA | 4 | 0.086 | 0.082 | 0.025 | 0.155 | 0.559 |  | 0.086 | 0.082 | 0.026 | 0.153 | 0.546 |  | 0.086 | 0.082 | 0.026 | 0.153 | 0.546 |

Table S2. AM fungal community data matrix with environmental variables. (TableS2.xlsx)


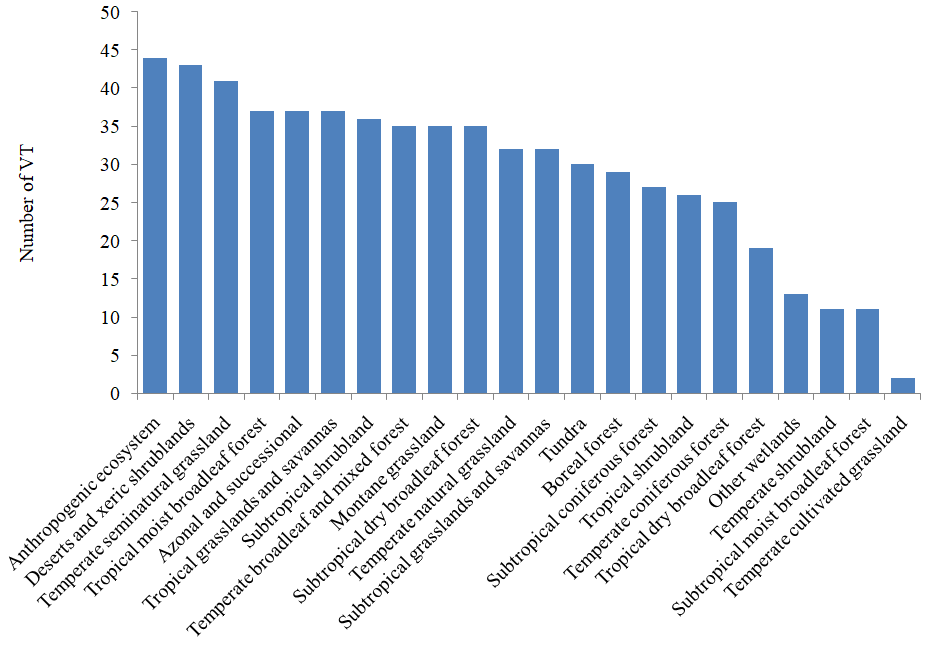
Figure S1. Biome occurrence worldwide of AM fungal virtual taxa (VT) recorded in this study. Bars indicate how many of the total of 49 recorded VT have previously been recorded in the biome types according to records from the MaarjAM database.

Figure S2. Biome occurrence worldwide of AM fungal virtual taxa (VT) recorded in this study. Bars indicate how many of the 22 biomes each of the 49 recorded VT has previously been recorded in according to records from the MaarjAM database.


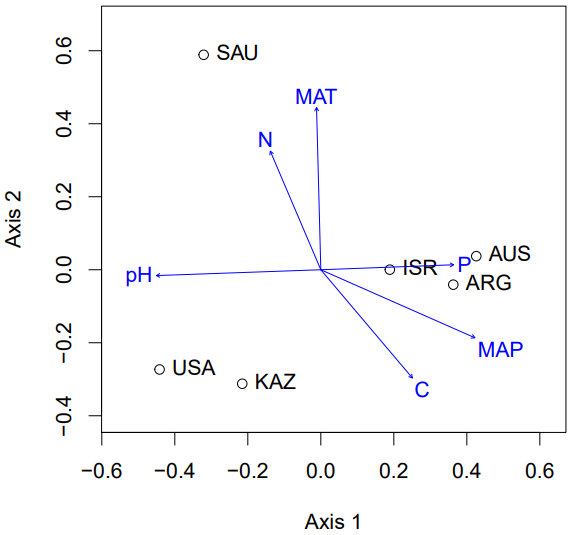


Figure S3. Two-dimensional principal coordinate analysis (PCoA) ordination plots, indicating variation in the composition of desert AM fungal communities. Arrows show significant correlations of climatic and soil-chemical parameters with the PCoA configuration. MAT – mean annual temperature, MAP – mean annual precipitation, C - soil organic carbon; N - total nitrogen; P – available phosphorus. ARG -Argentina, AUS - Australia, ISR - Israel, KAZ - Kazakhstan, SAU - Saudi Arabia, USA - United States of America.


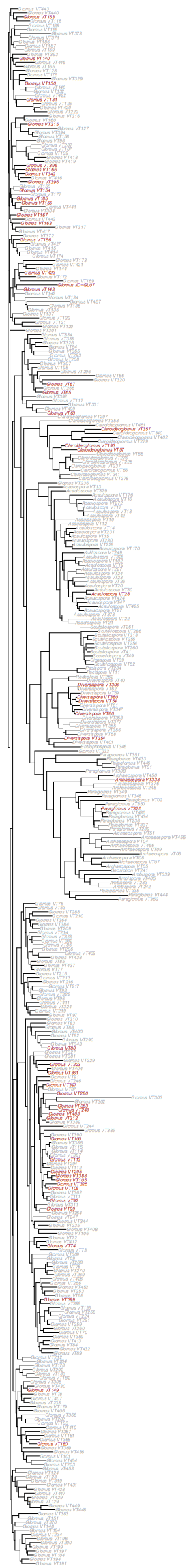


Figure S4. Neighbor-joining phylogenetic tree of full MaarjAM VT type sequences based on Jukes-Cantor distance. Phylotypes found in the desert samples are marked brown. (Figure S4.pdf)
