# Supplementary figures and images for: Arbuscular Mycorrhizal Fungal Communities in the Soils of Desert Habitats
Source: Microorganisms. 2021 Jan 22;9(2):229. doi: 10.3390/microorganisms9020229 (PMC7912695; doi:10.3390/microorganisms9020229)

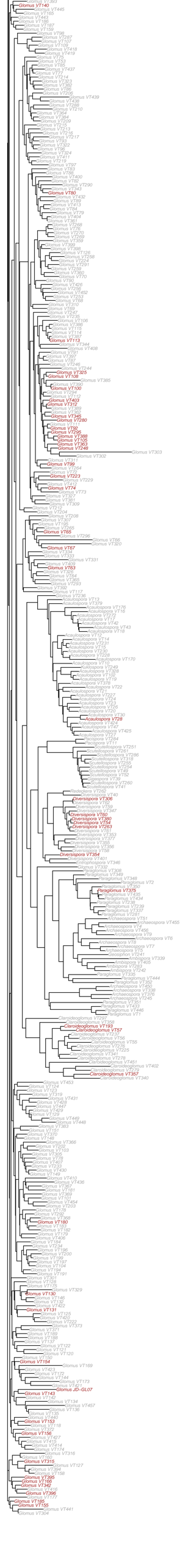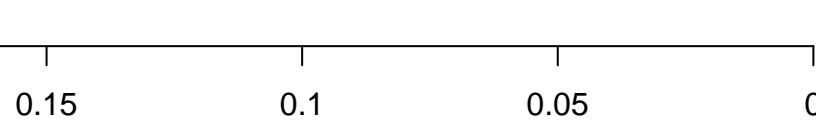

Supplement: Supplementary file 1 [file microorganisms-09-00229-s001.zip › Figure S4.pdf]
